# Spin effects in InAs self-assembled quantum dots

**DOI:** 10.1186/1556-276X-6-115

**Published:** 2011-02-03

**Authors:** Ednilson C dos Santos, Yara Galvão Gobato, Maria JSP Brasil, David A Taylor, Mohamed Henini

**Affiliations:** 1 Physics Department, Federal University of São Carlos, São Carlos, Brazil; 2 Physics Institute, UNICAMP, Campinas, Brazil; 3 School of Physics and Astronomy, Nottingham Nanotechnology and Nanoscience Centre, University of Nottingham, Nottingham, UK

## Abstract

We have studied the polarized resolved photoluminescence in an n-type resonant tunneling diode (RTD) of GaAs/AlGaAs which incorporates a layer of InAs self-assembled quantum dots (QDs) in the center of a GaAs quantum well (QW). We have observed that the QD circular polarization degree depends on applied voltage and light intensity. Our results are explained in terms of the tunneling of minority carriers into the QW, carrier capture by InAs QDs and bias-controlled density of holes in the QW.

## Introduction

Resonant tunneling diodes (RTDs) are interesting devices for spintronics because the spin character of the carriers can be voltage selected [1-4]. Furthermore, spin properties of semiconductor quantum dots (QDs) are also of high interest because electron spins can be used as a quantum bit [5] for quantum computing [6] and quantum communication [7]. In this paper, we have studied spin polarization of carriers in resonant tunneling diodes with self-assembled InAs QD in the quantum well region. The spin-dependent carrier transport along the structure was investigated by measuring the left- and right-circularly polarized photoluminescence (PL) intensities from InAs QD and GaAs contact layers as a function of the applied voltage, laser intensity and magnetic fields up to 15 T. We have observed that the QD polarization degree depends on bias and light intensity. Our experimental results are explained by the tunneling of minority carriers into the quantum well (QW), carrier capture into the InAs QDs, carrier accumulation in the QW region, and partial thermalization of minority carriers.

Our devices were grown by molecular beam epitaxy on a *n*+ (001) GaAs substrate. The double-barrier structure consists of two 8.3-nm Al_0.4 _Ga_0.6 _As barriers and a 12-nm GaAs QW. A layer of InAs dots was grown in the center of the well by depositing 2.3 monolayers of InAs. Undoped GaAs spacer layer of width 50 nm separate the Al_0.4 _Ga_0.6 _As barriers from 2 × 10^17 ^cm^-3 ^n-doped GaAs layers of width 50 nm. Finally, 3 × 10^18 ^cm^-3 ^n-doped GaAs layers of width 0.3 nm were used to form contacts. Our samples were processed into circular mesa structures of 400 μm diameter. A ring-shaped electrical contact was used on the top of the mesa for optical access and PL and transport measurements under light excitation. Magneto-transport and polarized resolved PL measurements were performed at 2 K under magnetic fields up to 15 T parallel to the tunnel current by using an Oxford Magnet with optical window in the bottom. The measurements were performed by using a Princeton InGaAs array diode system coupled with a single spectrometer. A linearly polarized line (514 nm) from an Ar^+ ^laser was used for optical excitation. Therefore, photogenerated carriers in the device do not present any preferential spin polarization degree. The right (σ^+^) and left (σ^-^) circularly polarized emissions were selected with appropriate optics (quarter wave plate and polarizer).

## Results and discussion

**Figure 1:**
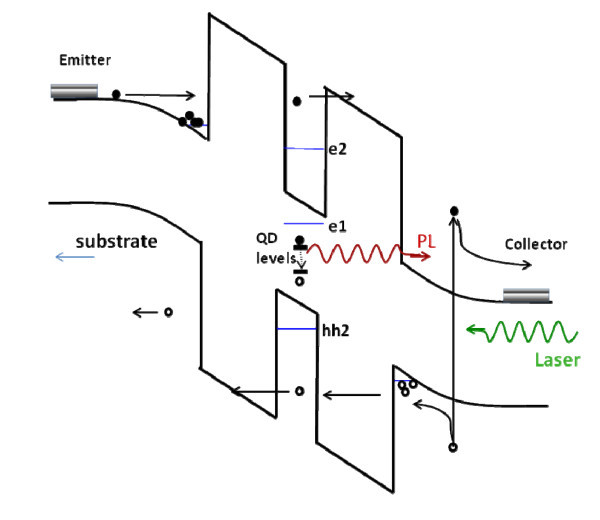
**Schematic potential profile and carrier dynamics in the RTD**.

Figure 1 shows the schematic potential profile and carrier dynamics in our device. Under applied bias voltage, electrons are injected from the GaAs emitter layer into the QW region. Resonant tunneling condition is obtained when the energy of carriers is equal to the energy of confined states in the QW. Under laser excitation, photogenerated holes tunnel through the QW and can be captured by the QDs and eventually recombine radiatively. Carrier capture into QDs occurs within typical times of about 1 ps which is much shorter than the characteristic dwell times of electrons and holes that are tunneling resonantly into the QW. Due to this fast carrier capture process, the QD photoluminescence will be very sensitive to the resonant tunneling condition and consequently to the applied bias voltage.

**Figure 2:**
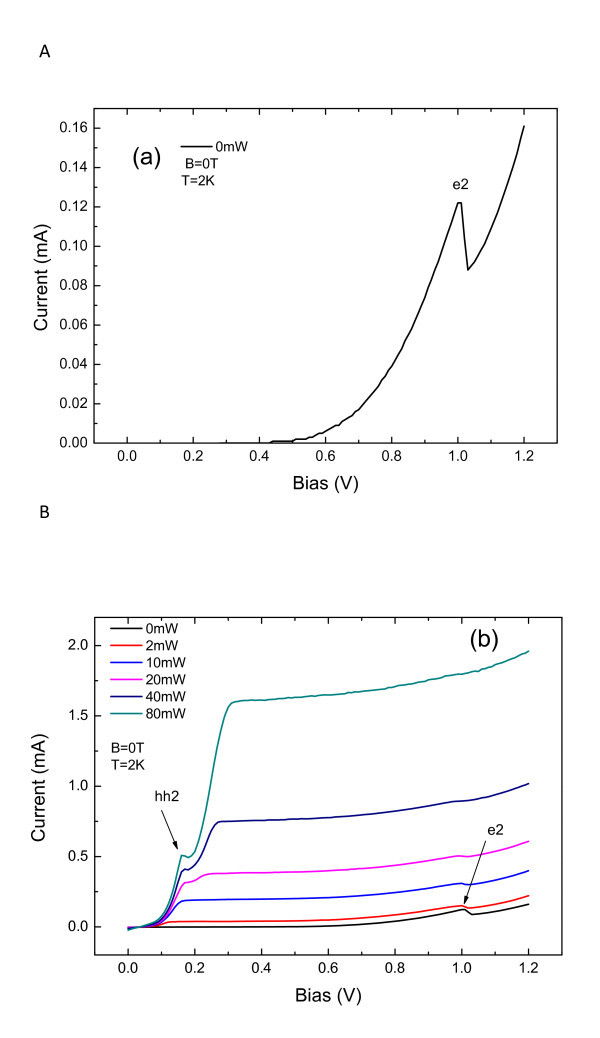
**Current-voltage characteristic curves**. (a) in dark and (b) for several laser intensities.

Figure 2 shows the I(V) characteristic curves for several laser intensities. In dark condition, we have observed only one electron resonant peak which was associated to the resonant tunneling through the second confined level e2 in the QW. It was shown previously [8] that even when QDs are formed, a wetting layer is still present and changes the position of the first QW confined level (e1) to a new position below the GaAs conduction-band. Therefore, resonant tunneling through e1 states cannot be observed in the I(V) characteristics curve. Under light excitation, holes are photocreated in contact layer region and tunnel through the double barrier structure. An additional resonant peak associated to hh2 hole resonance [8] is observed in lower voltage region under higher laser intensities. We have also observed that the photocurrent rapidly increases at low voltages (0.2 V), saturates in the region of about 0.2 and 0.4 V, and eventually follows the similar resonant voltage dependence as the current measured in dark conditions. We point out that even at zero bias, the QDs states which have a lower energy than the GaAs contacts, should be filled with electrons from the contact layers, resulting in a negative charge accumulation in the QW region. The potential profile of our structure should then be changed with respect to a reference sample without quantum dots [8,9]. In this case, an asymmetry in the impurity concentration of the contact layers should result in a non-zero electric field at the quantum well and, thus, in a non-zero current, at zero bias. We have indeed observed that the crossing of the I(V) curves under light excitation occurs at a voltage slightly larger than zero, which indicates that there is a small asymmetry in the impurity concentrations of the doped contact-layers. The crossing voltage corresponds to the flat band condition of the RTD structure with QDs.

**Figure 3:**
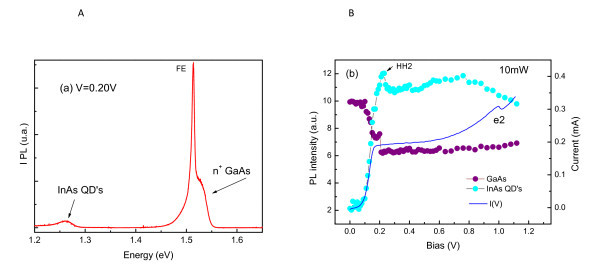
**Typical PL spectrum obtained and voltage dependence of PL intensity**. (a) Typical PL spectrum and (b) PL integrated intensity as a function of applied voltage at 2 K, for *B *= 0 T and 10-mW laser excitation.

Figure 3a shows a typical PL spectrum obtained under zero magnetic field (*B *= 0 T). The GaAs contact layers show two emission bands: the free-exciton transition from the undoped space-layer and the recombination between photogenerated holes and donor electrons from the n-doped GaAs layers. The QD emission is observed at about 1.25 eV and show lower PL intensity. We do not observe any emission from wetting layer because carriers preferentially recombine in lower energy states in QDs. We have also observed that the QD PL intensity depends strongly on the applied voltage at the region of low bias. We have observed a clear correlation between the I(V) curve and QD PL intensity (Figure 3b). Under applied bias, tunneling carriers can be promptly captured by QDs and then recombine radiatively. As explained before, due to this fast carrier capture process, the QD luminescence is sensitive to the resonant tunneling of carriers through the QW levels. Figure 3b also shows the voltage dependence of PL intensity from GaAs contact layer emission. Remark that QD and contact emission are in anti-phase with each other. The observed reduction of contact emission and increase of QD emission in low bias can be explained by the reduction of holes recombining in GaAs contact layer due to the efficient capture into the QDs [8,9].

**Figure 4:**
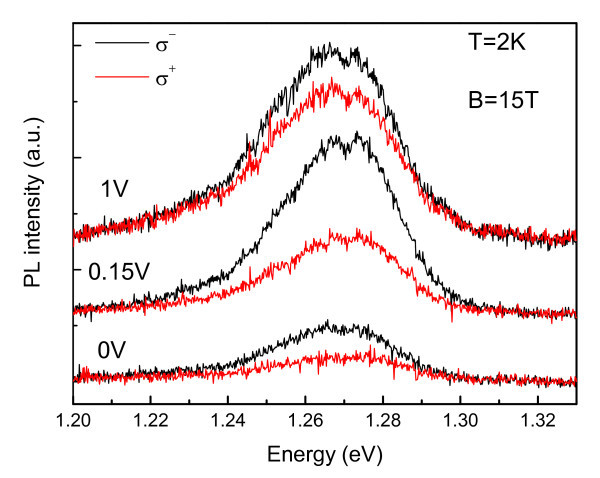
**PL spectra for different applied voltages at 15 T and 2 K**.

Figure 4 shows typical polarized resolved PL spectra from QDs under applied bias and magnetic field (15 T). Under magnetic field, the confined levels splits into spin-up and spin-down Zeeman states and the optical recombination can occurs with well defined selection rules probing the spin polarization of carriers in the structure [10,11]. We clearly observe that the relative intensities from σ+ and σ- QD emission bands vary with the applied bias voltage even though the spin-splitting of the QD PL emission is negligible and does not show any appreciable variation with the applied voltage. Therefore the observed spin splitting does not explain the voltage dependence of the QD polarization degree. In fact, the confined states of the QD should not follow a simple thermal equilibrium statistics, as the polarization of the carriers on those states should also depend on the polarization of the injected carriers, as we discuss below.

**Figure 5:**
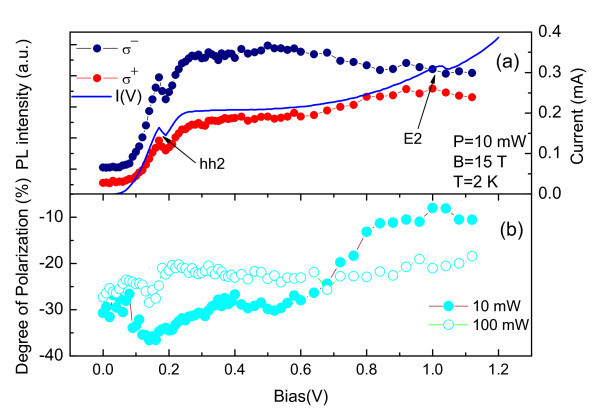
**Polarization of the injected carriers**. (a) Integrated PL intensity of QD emission as a function of applied voltage at 15 T. (b) Circular polarization degree of QD emission for lower and higher laser intensity as function of applied voltage at 15 T and 2 K.

Figure 5a shows the voltage dependence of the integrated PL intensity of QD emission at 15T. We have observed a good correlation between the I(V) curve and integrated PL intensity for the QD emission for both circular σ+ and σ- polarizations. Figure 5b shows the bias voltage dependence of the circular polarization degree for the QD emission under low and high laser intensities at 15T. We have observed that the QD circular polarization degree is always negative and that its value depends on both the applied bias voltage and the light excitation intensity. In general, its modulus presents a maximum value near the resonant tunneling condition for photo-generated holes. For the high laser intensity condition, the polarization of the QD PL band is nearly constant (~-25%), but it shows a clear bias voltage dependence for the low laser excitation intensity. In this case, the QD polarization degree clearly becomes more negative around the hole resonance and approaches zero at the electron resonance. Those results can be correlated to the density of carriers along the RTD structure and the electron and hole g-factors at the accumulation layer. We point out two basic information that are fundamental for this analysis. First, it is expected that the g-factors of electrons and holes have opposite signs for GaAs and second, the minority carriers tend to define the effective polarization of an optical recombination. Under high laser excitation intensity, the photocreated holes become the majority carrier for the whole bias voltage range of our measurements as demonstrated by the fact that the photocurrent due to photogenerated holes is markedly larger than the electronic current in dark. Therefore, the negative polarization of the QD emission should be mainly defined by the polarization accumulated electrons for all bias voltages, which is consistent with the g-factor for electrons in GaAs. Under low excitation condition, the majority carrier should change from holes at low voltages close to the hole resonant condition (hh2 resonant peak), to electrons at high voltages, close to the electron resonant condition (e2 resonant peak). Therefore, the QD polarization should be mainly defined by electrons at low voltages and by holes at high voltages, which explains that the negative polarization of the QD emission observed at low voltages tend to reduce its modulus and become more positive at high voltages.

Our results indicate that the final polarization from QD emission cannot be solely attributed to the spin-splitting of the QD states under magnetic field and it depends on the spin polarization of the injected carriers into the QW, which are determined by the g-factors and the density of electrons and holes along the RTD structure in a complex way. In fact, a quantitative calculation of the circular polarization degree from the QD emission is a rather complex issue as it depends on various parameters, including the g-factors of the different layers, the resonant and non-resonant tunneling processes, the capture dynamics of the carriers by the QDs, the density of carriers along the structure and the Zeeman and Rashba effects. This suggestion is also supported by previous results obtained for p-i-n and n-type RTDs without QDs [3,4]. It was observed that the high QW polarization degree observed on those measurements is mostly due to a highly spin polarized carriers from the two dimensional gas formed in the accumulation layer next to the emitter barrier. We also point out that the density of carriers along the RTD structure can be voltage and light controlled, which can be used to vary the circular polarization degree from QDs emission.

## Conclusion

In conclusion, we have observed that the QD circular polarization in an *n*-type RTD can be voltage and light controlled. A maximum value of spin polarization of about -37% was obtained for the hole resonant tunneling condition and for low-laser intensities. We associated this effect to the voltage and light dependence of charge accumulation in the QW region.

## Competing interests

The authors declare that they have no competing interests.

## Authors' contributions

EdS carried out the PL and transport measurements, prepared figures and participated in the analyses of the data. YGG conceived of the study, analyzed the data and wrote this paper. MJSPB participated in the draft of the manuscript. MH has grown the sample and DAT has processed the sample.
